# Juvenile emperor penguin range calls for extended conservation measures in the Southern Ocean

**DOI:** 10.1098/rsos.211708

**Published:** 2022-08-31

**Authors:** Aymeric Houstin, Daniel P. Zitterbart, Karine Heerah, Olaf Eisen, Víctor Planas-Bielsa, Ben Fabry, Céline Le Bohec

**Affiliations:** ^1^ Centre Scientifique de Monaco, Département de Biologie Polaire, Monaco 98000, Principality of Monaco; ^2^ Université de Strasbourg, CNRS, IPHC UMR 7178, Strasbourg F-67000, France; ^3^ Department of Physics, Friedrich-Alexander-University Erlangen-Nürnberg, Erlangen 91054, Germany; ^4^ Applied Ocean Physics and Engineering Department, Woods Hole Oceanographic Institution, Woods Hole, MA 02543, USA; ^5^ Zoophysiology, Department of Biology, Aarhus University, Aarhus C 8000, Denmark; ^6^ Alfred-Wegener-Institut, Helmholtz-Zentrum für Polar- und Meeresforschung, Bremerhaven 27570, Germany; ^7^ Fachbereich Geowissenschaften, Universität Bremen, Bremen 28359, Germany

**Keywords:** conservation biology, MPA network, polar regions, early life, seabirds

## Abstract

To protect the unique and rich biodiversity of the Southern Ocean, conservation measures such as marine protected areas (MPAs) have been implemented. Currently, the establishment of several additional protection zones is being considered based on the known habitat distributions of key species of the ecosystems including emperor penguins and other marine top predators. However, the distribution of such species at sea is often insufficiently sampled. Specifically, current distribution models focus on the habitat range of adult animals and neglect that immatures and juveniles can inhabit different areas. By tracking eight juvenile emperor penguins in the Weddell Sea over 1 year and performing a meta-analysis including previously known data from other colonies, we show that conservation efforts in the Southern Ocean are insufficient for protecting this highly mobile species, and particularly its juveniles. We find that juveniles spend approximately 90% of their time outside the boundaries of proposed and existing MPAs, and that their distribution extends beyond (greater than 1500 km) the species' extent of occurrence as defined by the International Union for Conservation of Nature. Our data exemplify that strategic conservation plans for the emperor penguin and other long-lived ecologically important species should consider the dynamic habitat range of all age classes.

## 1. Introduction

Anthropogenic environmental changes lead to upheaval even in remote and apparently untouched ecosystems such as the Antarctic and the Southern Ocean. Marine top predators, such as seabirds and marine mammals, play a pivotal role in marine ecosystems [1], and any disruptions in their abundance and distribution can have a major impact on the functioning and resilience of ecosystems [2]. At the same time, top predators are indicators of ecosystem health because of their high position in the trophic cascade and the vast, ocean basin-scale habitat of individual animals [3,4]. Thus, top predators integrate signals from across the food web and are therefore important bioindicators [5]. The health, abundance and distribution of marine top predators are consequently key metrics in ecosystem-based management and systematic conservation planning [6].

Effective conservation plans require comprehensive consideration of the at-sea distribution of species, including each life-history stage such as juveniles and immatures as they constitute an essential part of the total population [7]. However, in some of these taxa, in particular in many seabird species, the distribution of juveniles and immatures is difficult to assess and is therefore often neglected. This is especially true for polar ecosystems, where remoteness and the extreme environmental conditions hamper data collection.

Currently, the Southern Ocean is experiencing significant impacts owing to global change [8,9]. Measurable negative effects on wildlife have already occurred, such as population decreases of numerous seabird species [10,11], including the complete loss of emperor penguin (*Aptenodytes forsteri)* colonies [12,13]. The vanishing of these colonies has been attributed to strong El Niño events, rise in local mean annual air temperature, strong winds and/or decline in seasonal sea ice duration. Climate change is also expected to result in human access to new ice-free fishing areas [14], whereby seabirds and marine mammals will have to compete for food with industrial fisheries and may even become by-catch [15]. The accumulation of anthropogenic pressures on these fragile ecosystems urgently requires effective protection [16].

The Commission for the Conservation of Antarctic Marine Living Resources (CCAMLR) is the governing body in charge of conservation issues in the Southern Ocean. CCAMLR's mandate includes the implementation of conservation measures, such as the establishment of marine protected areas (MPAs) and the regulation of the fishing industry, through quota allocations and gear limitations [17]. Within the CCAMLR, conservation measures are based on the best scientific data available, including the distribution and demography of marine predators [18,19]. Similarly, the International Union for Conservation of Nature (IUCN)'s Red List of Threatened Species depicts the extent of occurrence (EOO) of each species, i.e. all the known, inferred or projected sites of present occurrence of the species' adults excluding cases of vagrancy [20]. Such knowledge serves then as a reference for policy making on the implementation of conservation measures. Consequently, providing novel data, in areas that have never been surveyed or on data-deficient population classes like juveniles enhances the conservation governance perspective for a species and its habitat.

Currently, 12% of the waters inside the CCAMLR boundaries have additional protection, with only 4.6% as no-take areas. This includes the Ross Sea and waters around the South Orkney Islands [16]. Since 2002, the CCAMLR has been working on establishing a network of MPAs around Antarctica, but the implementation of three new MPAs in east Antarctica [21], the Weddell Sea [22] and at the Antarctic Peninsula [23] has been difficult, proposals being under intense negotiations since 2010, 2013 and 2017, respectively [24,25]. However, even if implemented, the new MPAs would protect only 22% of the Southern Ocean inside the CCAMLR boundaries [16], which is significantly less than the IUCN recommended protection target of 30% of each marine habitat [26]. Furthermore, assessments and recommendations are based on limited and incomplete data. For instance, in the Weddell Sea, home to one-third of the global emperor penguin population [27], no tracking studies have been conducted so far; thus, very little is known about the penguins’ at-sea distribution in this area.

The emperor penguin is considered an iconic and ecologically important species of Antarctica. Its colony sites and at-sea movements have been the basis of previous discussions of conservation priorities, either in terms of MPAs [4], important bird areas [28,29] or areas of ecological significance [30]. With a population currently estimated at *ca* 270 000 breeding pairs in 61 known colonies around the continent [27], the species is severely threatened by global warming and expanding fishing activities in the Southern Ocean [15,31], facing the risk to be nearly extinct within this century [32]. The most effective actions to protect the emperor penguin from anthropogenic impacts would be a reduction in greenhouse gas emissions [31,32] as well as the establishment of MPAs throughout its habitat range [31]. Long-lived seabirds, emperor penguins reach sexual maturity between 4 and 8 years [33,34]. However, little is known about the first years at sea of the species, even though the survival of this age class referred as ‘juvenile’ is crucial for the viability of the global population [33,35]. To date, a total of only 48 juvenile emperor penguins have been tracked. Moreover, tracking has been done only in the Ross Sea and east Antarctica (table 1), even though for the designation of MPAs, it is fundamental to know their distribution at the circum-Antarctic scale [4,6,7].

**Table 1.** Tracking studies of juvenile emperor penguins at sea. (Colony details (location and size), tracking survey metrics (duration, distance and distribution) and proportion of the distribution area of tracked juveniles for each colony falling within the main oceanographic features (ACC and SO-TL) and conservation-related areas (IUCN, CCAMLR and MPAs) of the Southern Ocean. Single asterisk (*) means number of breeding pairs [27]. Double asterisk (**) means proportion of the distribution area falling within the mentioned feature. ACC: Antarctic Circumpolar Current; SO-TL: Southern Ocean Treaty Limits (i.e. at the parallel of 60° S as defined in the Antarctic Treaty); CCAMLR: Commission for the Conservation of Antarctic Marine Living Resources; IUCN-EOO: International Union for Conservation of Nature Extent of Occurrence (i.e. the EOO of the species considered by the IUCN [36]; MPAs: marine protected areas.)

colony	colony coordinates	colony population estimate*	no. birds tracked	mean tracking duration (days)	maximal distance from colony (km)	northernmost latitude reached	distribution area (millions km²)	%** in ACC area	%** in SO - TL	%** in IUCN - EOO	%** in CCAMLR area	%** in MPAs	publication
Cape Washington	74.58° S, 165.48° E	11 808	10	64	2845	56.9° S	1.7	54.6	73.5	14.6	73.5	4.4	Kooyman *et al*. [37]
Pointe Géologie	66.66° S, 140.00° E	2456	21	171	3503	53.76° S	3.6	75.9	62.7	35.8	98.0	13.5	Labrousse *et al*. [38]; Thiebot *et al*. [39]
Auster	67.38° S, 64.03° E	7855	10	121	2343	56.25° S	3.3	60.7	79.1	61.4	100	6.7	Wienecke *et al*. [40]
Taylor Glacier	67.47° S, 60.88° E	519	7	113	1570	54.23° S	1.7	87.3	67.7	43.4	100	9.3	
Atka Bay	70.62° S, 08.15° W	9657	8	221	2474	48.37° S	5.1	19.3	60.7	51.4	99.2	16.3	this study
mean	/	6459	11.2	138	2547	/	3.1	59.6	68.7	41.3	94.1	10.0	/
s.d.		4798	5.6	59.9	707.9		1.4	25.9	7.6	17.7	11.6	4.9	

**Figure 1.**
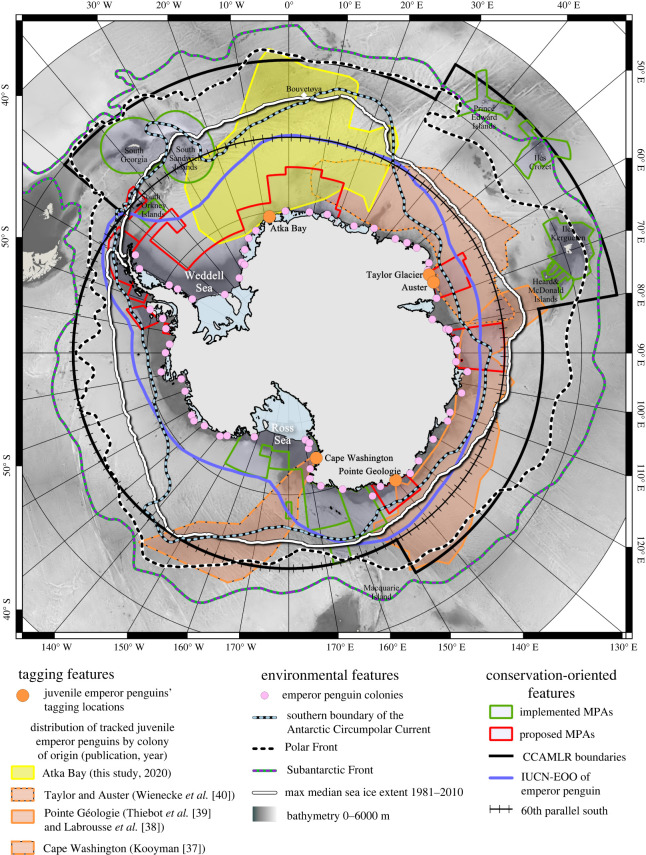
Overlap between existing and planned conservation zones and the distribution of juvenile emperor penguins tracked to date in the Southern Ocean. Distribution areas of juveniles are indicated by coloured polygons. MPAs: marine protected areas; CCAMLR: Commission for the Conservation of Antarctic Marine Living Resources; IUCN-EOO: International Union for Conservation of Nature Extent of Occurrence (i.e. the EOO of the species considered by the IUCN [36]).

The aim of this study was to bridge this gap in knowledge by equipping six-month-old emperor penguin chicks with ARGOS satellite platforms that transmit the birds' locations several times each day. Birds were tagged before their initial departure from their colony of origin at Atka Bay (70°37′ S, 08°09′ W) near the south eastern limit of the Weddell Sea (figure 1). We recorded their journey during their first year at sea (figure 1; electronic supplementary material, table S1). To assess the habitat range used by the juvenile emperor penguins at the scale of the Southern Ocean, we incorporated the distribution of all previously tracked juvenile emperor penguins into our analysis (figure 1; [37–40]).

## 2. Results

**Figure 2.**
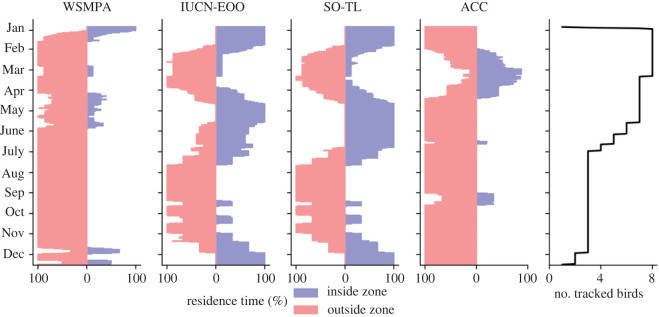
Proportion of time that the eight tagged juvenile emperor penguins from the Atka Bay colony spent either inside or outside the main conservation-related areas (WSMPA, IUCN-EOO) and oceanographic features (SO-TL, ACC) of the Atlantic sector of the Southern Ocean. For the ACC feature, inside refers to north of the southern boundary, while for WSMPA, IUCN-EOO and SO-TL, inside refers to their southern boundary, i.e. between their maritime boundary and the Antarctic continent. Daily average across all individuals computed over hourly data points. WSMPA: Weddell Sea marine protected area; IUCN-EOO: International Union for Conservation of Nature Extent of Occurrence (i.e. the EOO of the species considered by the IUCN [36]; SO-TL: Southern Ocean Treaty Limits (i.e. at the parallel of 60° S as defined in the Antarctic Treaty); ACC: Antarctic Circumpolar Current.

The tracking data from our study show that juvenile emperor penguins travelled north of 50° S (the lowest recorded latitude was 48.37° S), which is 600 km further north than previously recorded (table 1). Two of the eight tagged birds reached the South Sandwich Islands region in winter (late June until at least July) before their ARGOS platforms stopped transmitting. Thus, with three of the eight birds reaching sub-Antarctic areas, we can expect the presence of juvenile emperor penguins in these waters to be common rather than unusual. All tagged juveniles reached the southern boundary of the Antarctic Circumpolar Current (ACC), and five of the tagged birds remained in the ACC, i.e. between the southern ACC boundary and the Antarctic Polar Front, for prolonged time periods (greater than 46 days). One bird travelled north of the Polar Front [37,38]. The penguin tracks over a full year (polygon encompassing the area covered by the tracks; electronic supplementary material, figure S1) covered an area of 5.1 million km² (figures 1 and 2, table 1), nearly 1.4 times larger than the largest previously reported distribution of juvenile emperor penguins from their colony of origin (table 1).

Juvenile emperor penguins from the Weddell Sea area had a seasonal travel pattern similar to that of those tracked in other sectors of the Southern Ocean [37–40]. After leaving their colony, juveniles migrated northward towards and into the ACC where they remained for 37 ± 24 days. Juvenile emperor penguins commonly ranged outside the limits of the Southern Ocean (i.e. the parallel of 60° S as defined by the Antarctic Treaty, hereafter referred to as SO-Treaty); some birds travelled outside the CCAMLR boundaries (figure 1; electronic supplementary material, table S1). Over the course of April, juveniles migrated southward towards the pack ice to spend the winter (electronic supplementary material, figure S2).

Like in all other studies in which some fledglings have also been tracked long enough to record their northernmost extent [37–40], juvenile emperor penguins from the Weddell Sea area also swim all the way to the border of the Polar Front, even if the Polar Front is much further north than in east Antarctica and the Ross Sea. The extent of the north-south gradient appears to be the main factor driving the size of the distribution area (see Material and Methods). We assume that even if the duration of tracking was similar across studies, the absolute values of the distribution areas would differ but not their relative values.

In our study, juvenile emperor penguins spent only 51.1 ± 13.3% of their time inside the IUCN-EOO range of the species, which is based on the estimated adult distribution (electronic supplementary material, figure S2). Moreover, the time spent inside the area varied significantly across months (*p* < 1 × 10^−5^; figure 2). In August (winter), all penguins were outside and travelled up to 1260 km north of the IUCN-EOO, whereas they were mostly inside in January and May. When considering the data from all studies [37–40], 41.3 ± 17.7% of the observed distribution areas of juvenile emperor penguins fell within the IUCN-EOO (table 1). Juveniles from the Cape Washington colony in the Ross Sea travelled up to 1500 km outside the IUCN-EOO (table 1).

Taken together, the EOO of emperor penguins defined by the IUCN is underestimating the current habitat range of the species. Existing and planned MPAs cover on average only 10.0 ± 4.9% of the estimated distribution areas (table 1). Regarding the time spent inside protected areas, juvenile emperor penguins from the Atka Bay colony, which is located inside the proposed Weddell Sea MPA (WSMPA, the largest currently proposed MPA in the Southern Ocean), left the MPA's boundaries after 9 ± 4 days in January and remained only 10.6 ± 7.5% of their time inside the boundaries (figure 2). Only during summer (January and December) did the juveniles spend a considerable amount of time inside the WSMPA (47.9 ± 23.8% and 31.1 ± 13.4%, respectively). All tagged penguins were outside the WSMPA's boundaries in February and from July to November (figure 2).

## 3. Discussion

We find that juveniles travel beyond the boundaries of existing and planned conservation and management areas, demonstrating that conservation efforts in the Southern Ocean are insufficient to protect juvenile emperor penguins. Penguins are considered ecologically important species of the Southern Ocean's ecosystem [41]. Monitoring their population trend and distribution is therefore essential for biodiversity conservation. The common approach for designating boundaries of MPAs focuses on protecting the breeding segment of populations [42]. We argue that this might not be sufficient for species for which juvenile and adult ranges do not overlap, and we point out that the habitat range of juvenile penguins also requires a high level of protection. Indeed, juveniles are more vulnerable than adults as their foraging skills (including their ability to dive, to capture prey and to find productive feeding grounds) are not yet fully developed, and their experience to escape predators is minimal [43]. Moreover, juvenile survival can have a critical impact on the population dynamics, especially in long-lived species [44] as it is increasingly evidenced on various taxa (see [35,45–47]). Emperor penguins start breeding earliest at age 4–5 years, lay only one egg per pair and year, and only have an annual chance of 55% to bring a chick to fledging [32]. This low fecundity, projected to decrease under future warming scenarios [32,48], makes the survival of immature individuals, which represent about one quarter of the total population [33], particularly critical for the recruitment into breeding populations and thus the species' viability [49]. Moreover, in contrast with adults, the dispersal behaviour of juveniles is one of the main processes by which long-lived species will be able to adapt to the ongoing rapid environmental change. A vast travel range allows them to explore possible alternative feeding and breeding grounds [50]. In long-lived species, adult survival is the primary parameter influencing the population growth rate. However, reproduction can also significantly drive population dynamics and species with high adult survival can still have a declining population owing to an insufficient recruitment rate (i.e. juvenile survival to adulthood), see [35,45–47]. Therefore, for successful conservation, we need to consider the habitat range of all age classes.

Our findings reveal that juveniles commonly spent a substantial amount of time outside the species’ IUCN-EOO and outside the limits of existing or planned MPAs in the Southern Ocean (figure 1 and table 1; electronic supplementary material, table S1). Consequently, if protection measures were based solely on the current IUCN-EOO of the species, as it stands, given its focus on adult occurrences owing to the insufficient data for juveniles, this could lead to inefficient decisions for the future protection of the species. Furthermore, all studies including ours have reported that juveniles visit the highly productive ACC area during their first journey at sea, where the Antarctic Polar Front appears to act as an ecological barrier. During the most vulnerable stage of their life, the penguins' dispersive behaviour leads them outside the SO-Treaty and CCAMLR limits into waters where they are likely to encounter and compete with fisheries (see [29,51] for current data on fisheries activity). In addition, overlap with fisheries in the Weddell Sea region might occur in forthcoming decades and increase the threat to emperor penguins according to the projected long-term effects of climate change on krill population migration [24,52]. In accordance with the CCAMLR's ecosystem-based fisheries management approach, the presence of this critical fragment of the emperor penguin population should be considered by the CCAMLR when allocating fishing quotas and zones; especially in the current context where several CCAMLR fishing states are lobbying for an increase of the spatial and temporal distribution of catches and fisheries [53].

A growing body of evidence indicates the ongoing threats to penguins. Trathan *et al*. [31] recently advocated for a reclassification of the emperor penguin on the IUCN Red List from the current ‘Near Threatened’ status to ‘Vulnerable’ or ‘Endangered’, together with the classification as an ‘Antarctic Specially Protected Species' by the Antarctic Treaty. Our data support this call for better protection by also pointing out the need to include all age classes and age-specific threats into the classification assessment [4,7].

Indeed, ignoring young age classes, their higher vulnerability, and the critical impact of immature survival and recruitment parameters on the population dynamics, would lead to biased estimates of overlap with threats and may misdirect management and conservation efforts. However, owing to the limited datasets in space (individuals of 8% of the colonies have been tracked for now) and time (no multi-year datasets except for Pointe Géologie colony), additional monitoring studies on juvenile emperor penguins need to be conducted to improve their surveillance. Yet, the consistency in their behaviour despite the diversity of environments tracked birds belong to, allow speculation that juvenile emperor penguins make use of a large part of the offshore Antarctic Convergence area at the circumpolar scale, where they also overlap with juvenile king penguins (*Aptenodytes patagonicus*) [54]. Including widely ignored juvenile tracking data of these Southern Ocean sentinels into synthetic approaches to designate high priority conservation areas [4] will enhance the identification and delineation of conservation hotspots. This is essential to the creation of a well-designed network of ecological areas [55] that would provide a more robust protection to the Southern Ocean ecosystem [16].

## 4. Material and methods

### 4.1. Study site and instrumentation

Our study was conducted at the Atka Bay emperor penguin colony (70°37′ S, 08°09′ W) near (approx. 10 km) the German Antarctic research base ‘Neumayer Station III’. In January 2019, we equipped eight 6-month-old chick emperor penguins with satellite communicating SPOT-367 ARGOS platforms (Wildlife Computers, Redmond, WA 98052, USA) [56]. The ARGOS platforms were programmed to transmit their identification every day at 4.00, 6.00, 10.00, 16.00, 19.00 and 21.00, corresponding to time points with optimum ARGOS satellite coverage over the Weddell Sea (ARGOS CLS, Toulouse, France).

To minimize drag, the ARGOS platforms were deployed on the lower back of the birds [57,58]. The streamlined devices were attached to the feather with adhesive tape (Tesa tape 4651, Beiersdorf AG, Hamburg, Germany) and secured with three cable ties (Panduit PLTM1.5M-C0 142*2.6 mm, Panduit Corp, Illinois, USA). We then applied epoxy glue (Loctite EA 3430, Loctite, Henkel AG., Düsseldorf, Germany) on the mounting to increase waterproofing and robustness [59,60].

### 4.2. Estimation of the at-sea distribution of juvenile emperor penguins from the Atka Bay colony

#### 4.2.1. Location filtering

ARGOS locations are associated with spatial error ellipses. These spatial errors can range from a few hundred meters to several kilometres [61,62]. Erroneous locations were filtered out using a speed filter from the R package ‘*argos filter*’ [63] with the maximum travel speed fixed at 15 km h^−1^ following similar studies on emperor penguins [38,64].

#### 4.2.2. Interpolation of locations at a regular time step

We used a state-space modelling approach [65] to estimate hourly locations. Specifically, a Kalman filter, which accounted for location error, was applied using the R package ‘*crawl*’ [66], and continuous-time correlated random walk models were used to predict locations at a regular time-step interval of 1 h [65,67].

### 4.3. Estimation of the colony-specific distribution area for juvenile emperor penguins

In addition to the eight birds tracked in our study, 48 juvenile emperor penguins from four different colonies were previously tracked ([37–40], table 1 for the details on the colonies). Data of these previously acquired bird journeys are available as maps in the respective publications. We georeferenced these tracking maps using the QGIS software. We subsequently plotted the main corner points encompassing the tracks of all birds from each colony (electronic supplementary material, figure S1). We obtained the distribution of juvenile emperor penguins by computing the concave hull envelope for each dataset using the ‘*ConcaveHull’* plugin [68]. Envelopes from the same colony [38,39] were merged to consider only one polygon per colony (referred to as distribution area), including one for the Atka Bay colony. The size of each distribution area was calculated with the ‘*raster’* package in R [69] and is reported in table 1. Owing to the significant overlap of Auster and Taylor Glacier juvenile distribution [40] and the proximity (132 km) of the two sites [70], for visualization purposes, the tracks of the birds from Auster and Taylor Glacier colonies are shown in the same polygon in figure 1. However, the distribution areas were computed separately for each colony.

### 4.4. Ecological features

The locations of the Southern Ocean fronts and the ACC boundaries [71] were downloaded from https://gis.ccamlr.org [72].

The bathymetry at 1 min horizontal spatial resolution was obtained from the ETOPO1 Global Relief Model provided by the NOAA National Geophysical Data Center [73].

Sea ice concentrations (ranging from 0 to 100%) were obtained from Advanced Microwave Scanning Radiometer (AMSR-2) satellite estimates of daily sea ice concentration at 3.125 km resolution from the University of Bremen (https://seaice.uni-bremen.de/data/amsr2/) [74]. The sea ice edge contour was defined by the 15% sea ice concentration [75,76] (electronic supplementary material, figure S2).

The maximum and minimum median sea ice extent from 1981 to 2010 presented in the electronic supplementary material, figure S1 and figure S2 were obtained from the National Snow and Ice Data Center NSIDC [77] implemented in the ‘*Quantarctica3*’ package [78] of the QGIS software.

### 4.5. Conservation-oriented features

The CCAMLR planning domains and existing Antarctic MPAs were obtained from https://gis.ccamlr.org [72]. The proposed WSMPA [22] and the proposed east Antarctic MPA boundaries [21] were obtained from www.mpatlas.org [79]. The Domain 1 MPA proposal [23] was drawn from www.mpatlas.org [79]. The South Georgia and South Sandwich Islands MPA and the sub-Antarctic MPAs boundaries were downloaded from www.protectedplanet.net.

The IUCN-EOO of the Emperor penguin species was obtained from www.iucnredlist.org [36].

### 4.6. Assessing the overlap between bird distribution and conservation-oriented areas

The average residency time that each of the birds equipped in our study spent inside existing or proposed conservation-oriented areas of the Southern Ocean was computed on a daily, weekly or monthly basis, or averaged over the total tracking period.

We tested whether the observed monthly averaged residency time changed significantly over the course of a year using the Kruskal–Wallis rank sum tests. For all tests, the significance threshold was set at *p* = 0.05. Statistical analyses were performed using the software R v. 3.5.0 [80] and QGIS v. 2.18.18 [81] with the data package ‘*Quantarctica3*’ [78].

## Data Availability

The dataset generated and analysed during this study is available in the Movebank Data Repository, https://doi.org/10.5441/001/1.sg2s0f7q [82]. Data is also available in the electronic supplementary material [83].
